# The Effect of Short-Term Training Course by Nurses on Body Image in Patients with Burn Injuries

**DOI:** 10.29252/wjps.8.3.359

**Published:** 2019-09

**Authors:** Mahnaz Seyedoshohadaee, Alice Khachian, Naimeh Seyedfatemi, Mokhtar Mahmoudi

**Affiliations:** 1 Department of Medical Surgical Nursing, School of Nursing and Midwifery, Iran University of Medical Sciences, Tehran, Iran;; 2 Department of Psychiatric Nursing, School of Nursing and Midwifery, Iran University of Medical Sciences, Tehran, Iran

**Keywords:** Burns, Psychology, Body image, Training course

## Abstract

**BACKGROUND:**

Burn is the fourth most common cause of trauma worldwide and is the fourth among the injuries and surgeries requiring medical care. This study was designed to determine the effect of a short-term training course by nurses on body image in patients with burn injuries.

**METHODS:**

This study was a semi-experimental single-group survey assessing before and after interventions. Totally, 130 subjects (65 women and 65 men) were enrolled. The training course was held in 3 sessions of two hours. The contents of the group training course (a group of 5 people) were in relation to the body image and the factors affecting it, and the ways to improve the body image. The data collection instrument was the satisfaction with appearance scale (SWAP).

**RESULTS:**

The mean scores of the body image of patients before and after the intervention were 49.44±11.39 and 41.63±11.89, respectively. There was a significant difference between the mean scores of body image before and after educational interventions (T=6.013, P≤0.001). The mean score of body image in women before and after intervention was 49.2±10.9 and 41.2±11.65, respectively (T=4.51, P≤0.000). The mean score of body image in men before and after intervention was 49.6±11.89 and 42.07±12.19, respectively (T=4.51, P≤0.000).

**CONCLUSION:**

Short-term courses held by nurses were shown to have a significant role in improving the body image of burn patients.

## INTRODUCTION

Burn is the fourth most common cause of trauma worldwide and is the fourth among the injuries and surgeries requiring medical care.^1^ Today, the development of medical sciences, including plastic surgeries, fluid therapy and intensive care of burn patients has resulted in increase of the survival of many of burn patients who have never had a chance to survive in the past.^2^ This has increased the need for long-term care for these patients.^3^ Deformities caused by burns, particularly in apparent areas, have led to a tension in the injured person as well as a significant change in the performance and behaviors.^4^^,^^5^

One of these disorders is a disturbance in the body image.^6^ The body image is a set of individuals’ self-conscious and unconscious attitudes toward their own bodies, past attitudes and perceptions, and it contains their feelings about their bodies, functions, appearances and physical capacities, as well. The body image is the perceptions, emotions and thoughts that individuals have about their own body appearances.^7^ The individuals’ feelings about the bodies and appearances have important implications for the assessment and treatment of the patients as a whole and because of this, the importance of individuals’ emotions towards their appearances and bodies is emphasized in the care and treatment of the mentioned patients.^8^

Today, there is an interest in the assessment of the patients’ body image who has appearance changes or deformity as a result of a disease, because apparent changes can have significant effects on self-esteem and body image, and consequently on the continuation of life after it.^4^ The body image plays a significant role in determining the quality of a person’s life^9^ and dissatisfaction with the body image has negative effects on the physical and mental dimensions of the quality of life.^10^ Considering the importance of the body image in burn patients, many researchers are interested in this regard. In a qualitative study conducted in 2013, Hodder *et al.* studied the role of social support in burn patients and its impact on their body images. In this study, nine adult women participated and each identified four items that affected their body images. These items included acceptance, social comparison, talking about appearance concerns, and the gaze of others.^11^

In another study conducted in 2014, Lam *et al.* showed that the body image of burn patients is very significant and it is recommended that for a better adaption of these people with their problems, care and treatment interventions should be undertaken at the right time to prevent psychological problems in long term.^12^ In another study conducted recently, Ajoudani *et al.* studied the social support, community presence, and body image in burn patients. In this study, 100 patients were followed up for 1 year after discharge. The results of this study showed that the body image in these patients was significantly influenced 6 months after discharge. These results also demonstrated that there was a correlation between these three variables.^13^

In another study conducted in Iran, Niroumand-Zandi *et al.* studied the satisfaction of the body image and the factors affecting it in burn patients. The results of this study showed that social support in family, place of burn, burn severity, marital status and age are factors that affect the satisfaction of the body image.^14^^-^^17^ Although, the body image in burn patients and its effects on other aspects of the patients are very important, little research has been conducted in relation to the ways to improve body image in these patients, while it has always been mentioned about the importance of improving the body image of these patients. Therefore, based on the importance of this issue and the limited studies in this regard, the present study was designed with the aim of studying the effects of short-term training courses by nurses on the body image in patients with burn injuries.

## MATERIALS AND METHODS

This study was a semi-experimental single-group survey conducted in Shahid Motahari Burns Hospital affiliated to Iran University of Medical Sciences and assessed the before and after interventions. The enrolled population were women and men aged 18 years and older and had burns (above 15%), and were alert without any history of known psychological diseases, and all were able to answer questions and had been admitted for at least three days. People who were burned for suicide were not enrolled in the study. The sample size was estimated using the results of other studies with 95% confidence, and 80% test power, while the sample drop was 10%. Finally, 130 patients (65 women and 65 men) were enrolled. 

After obtaining informed consent from the participants, and receiving official permission and ethical code (ethics code=987) and obtaining permission from the relevant authorities and coordinating with the hospital departments, the researcher introduced himself to the research units and explained the research objectives and how to complete the questionnaire. Thirty minutes was spent to complete each questionnaire. After completing the questionnaires and calculating the mean scores for patients with lower grades, the training course was held for men and women separately in 3 sessions of two hours (a total of 6 hours for women and 6 hours for men). The contents of the group training course (a group of 5 people) were in relation to the body image and the factors affecting it, and the ways of improving the body image. 

At the end of the course, the content was given in a small handbook to the participants. A month after the workshop, body image of the patients was re-measured and compared with pre-intervention scores. All stages of the study were done by the nurses. The data collection instrument in this study was the satisfaction with appearance scale (SWAP) conducted by Lawrence *et al.* (1998). SWAP was a 14-item questionnaire in order to evaluate the body image of burn patients and was introduced to the burn center in 1998. This questionnaire was designed to assess the satisfaction of the body image in burn patients. The internal consistency was estimated as α=0.87 in patients with burn injuries.^18^

This questionnaire was used to examine the body image in people with deformities like scleroderma. The questions were divided into two sections. Perceived social impact contained 6 questions and subjective dissatisfaction scale contained 8 questions. Each question was responded on a 7-point Likert, ranging from completely disagree (0) to completely agree.^7^ Scoring in questions 4-11 was in a reverse manner. The minimum score in this scale was zero and the maximum was 84. A higher score indicated a greater dissatisfaction with the body image. In this study, the mean scores of women and men with burn injuries before and after the training course were compared with each other.^4^^,^^18^^,^^19^ Data were analyzed using the Statistical Package for the Social Sciences (SPSS, Version 20, Chicago, IL, USA) and descriptive and inferential statistics were performed to analyze the data.

## RESULTS

The mean age of the participants was 37.3±9.6 years (the mean age in men and women was 37±10.3 and 37.6±9, respectively). In terms of burn percentage, the mean burn severity was 31.7±7.13 percent. Table 1 shows the demographic data of patients participating in this study with more details. The mean scores of the body image of the patients before and after the intervention were 49.44±11.39 and 41.63±11.89, respectively. Based on the results of paired sample t-test, there was a significant difference between the mean scores of body image before and after educational interventions (T=6.013, P≤0.001).

**Table 1:** Demographic characteristics of participants

**Sex**	**Men**		**Women**		**Total**	
**Burn severity**	**NO.**	**Percent**	**NO.**	**Percent**	**NO.**	**Percent**
10-20	0	0	1	1.5	1	0.8
21-30	30	46.2	28	43.1	58	44.6
31-40	27	41.5	26	40	53	40.8
41-50	8	12.3	10	15.4	18	30.8
Total	65	50	65	50	130	100
Mean±SD	31.7±7.13 MAX=47, MIN=20					
**Sex**	**Men**		**Women**		**Total**	
**Age**	**NO.**	**Percent**	**NO.**	**Percent**	**NO.**	**Percent**
20-30	21	32.3	14	21.5	35	26.9
31-40	17	26.2	25	38.5	42	32.4
41-50	21	32.3	20	30.8	41	31.5
≥50	6	9.2	6	9.2	12	9.2
Mean±SD	37±10.3		37.6±9		37.6±9.3	
Domain	20-35		20-54		20-54	
Total	65	50	65	50	130	100
Employed	41	63	40	61.5	81	62.3
Unemployed/Housewife	24	37	25	38.5	49	37.7
Total	65	50	65	50	130	100

Table 2 shows the mean scores of body image in patients before and after educational interventions with more details. The results, when the mean body image before and after educational interventions was analyzed by sex, showed that the mean scores of body image in women and in men after intervention to be significantly higher than before the intervention. The mean score of body image in women before and after intervention was 49.2±10.9 and 41.2±11.65, respectively (T=4.51, P≤0.000). The mean score of body image in men before and after intervention was 49.6±11.89 and 42.07±12.19, respectively (T=4.51, P≤0.000).

**Table 2:** Frequency distribution of patients’ body image scores before and after intervention

**Patients’ body** **image scores**	**Before**		**After**	
	**No.**	**Percent**	**No.**	**Percent**
0-28	4	3	15	11.5
29-57	91	70	103	79.2
58-86	35	27	12	9.3
Total	130	100	130	100
Mean	49.44±11.3		41.63±11.8	
Results	T=6.013, P≤0.000			

## DISCUSSION

Changes in the individuals’ body image has been identified as one of NANDA’s nursing diagnosis in 1973 and nurses must plan an exclusive nursing care for each patient due to changes in the body image. Patients with extensive burns are a group of patients who are at high risk of impaired body image. The present study was conducted to study the effects of a short-term training course by a nurse on the body image in patients with burn injuries. The results of the present study demonstrated that short-term training course significantly affected the body image of these patients. The advantage of this study included courses held by nurses. Another benefit of this study was the short duration of the courses, because it was provided for patients more practical and in an easier way. 

Although several studies have pointed to the importance of body image and its promotion in patients with burn injuries, our searches did not show any study to assess the effect of educational interventions on improving the body in this group of patients. However, studies that were almost similar to the present study were cited. The results of all these studies indicated that the provision of educational services to burn patients could improve the status in a variety of aspects. In a study conducted in 2017, Li *et al.* denoted to the effects of a nursing program, including enhanced social support, intensive health education, comprehensive psychological intervention and graded exercise in patients with burn injuries.^20^

In this study which lasted 5 weeks, the patients were divided into two groups of 30 patients and those who were in the intervention model group received the intervention program. The results of Lin *et al.* showed that the intervention group had significantly better scores than the control group for physical function, psychological function and social function.^20^ In another study, the effects of multimedia education on the quality of life in patients with burn injuries were studied. In this study, 100 patients were evaluated. Fifty patients received the routine care and the other 50 received the multimedia training packages in addition to routine care. The quality of life in patients before and 3 months after intervention were examined. The results of this study demonstrated that the quality of life in patients who were in the intervention group significantly improved 3 months after receiving the educational package compared to the control group.^21^

Treatment and care of patients with intensive burns usually takes time and requires teamwork.^22^^,^^23^ However, in many cases in this regard, the role of nurses is neglected, while nurses can play a major role in rehabilitation of these patients. This issue is seen more widely among developing countries like Iran. In most cases, Iranian nurses only work in the acute phase of the treatment of patients with intensive burns, and their role in the rehabilitation process is very limited and insignificant. The importance of nurses’ role in rehabilitation of intensive burns in developing countries is twofold due to the high incidence of burns and the limited financial and human resources. One of the effective actions that nurses can do is to provide educational services for this group of patients.^24^

As the results of the present study showed, holding short-term training courses by nurses can play a significant role in improving the body image of burn patients. This study has some limitations. One of these limitations is that the study was a single-group study. The short follow-up period is another limitation of this study. Therefore, it is recommended that further studies to be conducted considering a control group and a longer follow-up period. The body image of individuals with burn injuries can affect the treatment process and their survival. The provision of treatment and care regarding this is very important. The results of this study showed that short-term courses held by nurses had a significant role in improving the body image of burn patients. Due to the extreme limitations of studies, it is recommended that more similar studies to be conducted in this regard. It is also recommended that the effects of short-term training by nurses on other aspects, such as physical problems and psychological disorders to be considered as well.
